# *ExVe*: The knowledge base of orthologous proteins identified in fungal extracellular vesicles

**DOI:** 10.1016/j.csbj.2021.04.031

**Published:** 2021-04-17

**Authors:** Vinícius da Silva Coutinho Parreira, Letícia Graziela Costa Santos, Marcio L. Rodrigues, Fabio Passetti

**Affiliations:** a Instituto Carlos Chagas, FIOCRUZ, Rua Prof. Algacyr Munhoz Mader, 3775, CEP 81350-010, Curitiba/PR, Brazil; b Instituto de Microbiologia Paulo de Góes, Universidade Federal do Rio de Janeiro (UFRJ), Brazil

**Keywords:** Extracellular vesicle, Fungi, Orthology, Shotgun proteomics

## Abstract

Extracellular vesicles (EVs) are double-membrane particles associated with intercellular communication. Since the discovery of EV production in the fungus *Cryptococcus neoformans*, the importance of EV release in its physiology and pathogenicity has been investigated. To date, few studies have investigated the proteomic content of EVs from multiple fungal species. Our main objective was to use an orthology approach to compare proteins identified by EV shotgun proteomics in 8 pathogenic and 1 nonpathogenic species. Using protein information from the UniProt and FungiDB databases, we integrated data for 11,433 hits in fungal EVs with an orthology perspective, resulting in 3,834 different orthologous groups. OG6_100083 (Hsp70 Pfam domain) was the unique orthologous group that was identified for all fungal species. Proteins with this protein domain are associated with the stress response, survival and morphological changes in different fungal species. Although no pathogenic orthologous group was found, we identified 5 orthologous groups exclusive to *S. cerevisiae*. Using the criteria of at least 7 pathogenic fungi to define a cluster, we detected the 4 unique pathogenic orthologous groups. Taken together, our data suggest that Hsp70-related proteins might play a key role in fungal EVs, regardless of the pathogenic status. Using an orthology approach, we identified at least 4 protein domains that could be novel therapeutic targets against pathogenic fungi. Our results were compiled in the herein described ExVe database, which is publicly available at http://exve.icc.fiocruz.br.

## Graphical abstract

**Figure:**
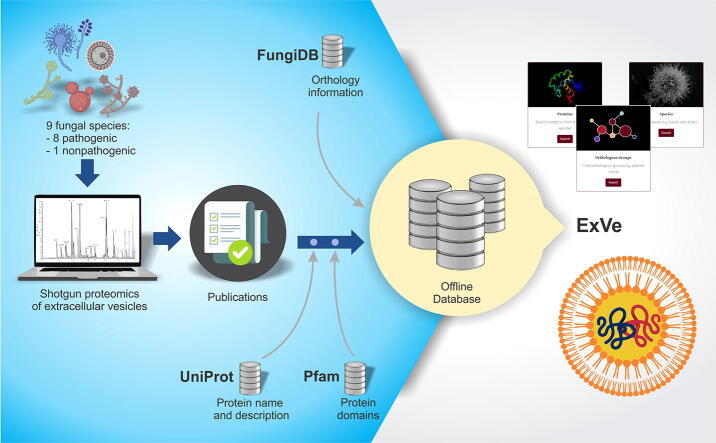

## 1 Introduction

Eukaryotic and prokaryotic organisms release small bilayer extracellular vesicles (EVs) ranging from 20 to 5,000 nm under distinct biological or environmental conditions [1], [2], [3]. In eukaryotes, EVs is a generical term referring to bilayered membrane structures that are released by many cell types. EVs are classified into exosomes, microvesicles, and apoptotic bodies based on size, cargo and biogenesis pathways [4]. Exosomes are small structures in the 30 to 100 nm diameter range generated from the release of luminal vesicles in late endosomes after fusion with the plasma membrane [1], [5]. Microvesicles range from 100 nm to 1000 nm in size, and they are formed by budding off the plasma membrane [5], [6]. Apoptotic bodies are the largest vesicle structures (1 µm to 5 µm) [1], observed only in cells that undergo apoptosis [1], [5].

The functions of EVs are consistently associated with cellular communication. EVs are known to transport several molecules to distant organs or tissues [4], [7]. In fungi Rodrigues *et al*. (2007) described that EV plays a role in fungal *trans*-cell wall transport [8]. Monguió-Tortajada and colleagues (2017) identified an immunomodulatory potential of EVs derived from umbilical cord mesenchymal stem cells [9]. Cancer cells also produce EVs, which are related to tumor development and progression [10], invasion [11], angiogenesis [12] and metastasis [13]. In pathogens, EVs are the vehicles of exportation of several antigens [14], [15], [16]. Pathogen-derived EVs also participate in the host-pathogen interaction [8], [14], [17], since they concentrate virulence factors [4], [18], [19], [20], [21], [22]. The role of EVs as vehicles of virulence factors for many taxa, including fungi, protozoa and bacteria, has been comprehensively reviewed by Campos and colleagues [7].

The seminal discovery of EV production in the fungal pathogen *Cryptococcus neoformans* unraveled a mechanism of secretion in fungal cells that was extended to other fungi [4], [8]. For instance, Ikeda and colleagues (2018) associated *Sporothrix brasiliensis* EVs with host environmental adaptation and increased fungal pathogenicity. *Sporothrix* EVs contained heat shock proteins, major facilitator superfamily transporters, and other enzymes that could increase fungal virulence [23]. *Candida albicans* EVs were described as playing a role in biofilm drug resistance. Additionally, iRNA sequences in *C. albicans* EVs have the potential to modulate gene expression in host cells [24], [25]. In *Cryptococcus* EVs contain several molecules associated with virulence and survival in the host environment, including laccase, glucosylceramides and urease [26]. Several other examples of the biological functions of fungal EVs are available in the current literature, as recently reviewed by Rizzo and colleagues [17].

The molecular composition investigation of fungal EVs is the first step towards the comprehension of their functional role in pathogenic and nonpathogenic species. Among others, the description of EV proteins is currently the focus of several research groups. These studies can benefit from the new proteomic technologies and facilitated EV isolation methods [27], [28], [29]. An accurate method of EV isolation associated to an efficient proteomic shotgun approach may contribute to the identification of EV-associated proteins in a large number of pathogenic and nonpathogenic fungal species [4], [8], [20], [30].

The integration of publicly available datasets is a trend in modern science to accelerate novel findings. Due to the large amount of data, databases focused on gathering and organizing this information may contribute to data sharing and encourage other studies [18], [31], [32]. ExoCarta [33], Vesiclepedia [34], and EVpedia [35] are the databases currently available to investigate EV proteomics data of several species. These databases may assist researchers in data mining of published data and additional analysis of their own datasets [18], [33], [34], [35]. However, there is no publicly available database regarding EV data of fungal species [35].

To integrate gene and protein data of distinct fungal species, orthology databases have been created [36], [37]. Orthology analysis may contribute to understanding the biological function of different proteins and biological pathways under many conditions and even to comprehending the evolutionary history of a group or species [31], [37]. To date, there is no publicly available database to integrate proteomics data from pathogenic fungal EVs. Here, we present ExVe, a publicly available database that integrates EV proteomic data from nine fungal species focusing on orthology, which can be freely accessed at http://exve.icc.fiocruz.br.

## 2 Material and methods

### 2.1 Fungal EV proteomics data

**Fig. 1:**
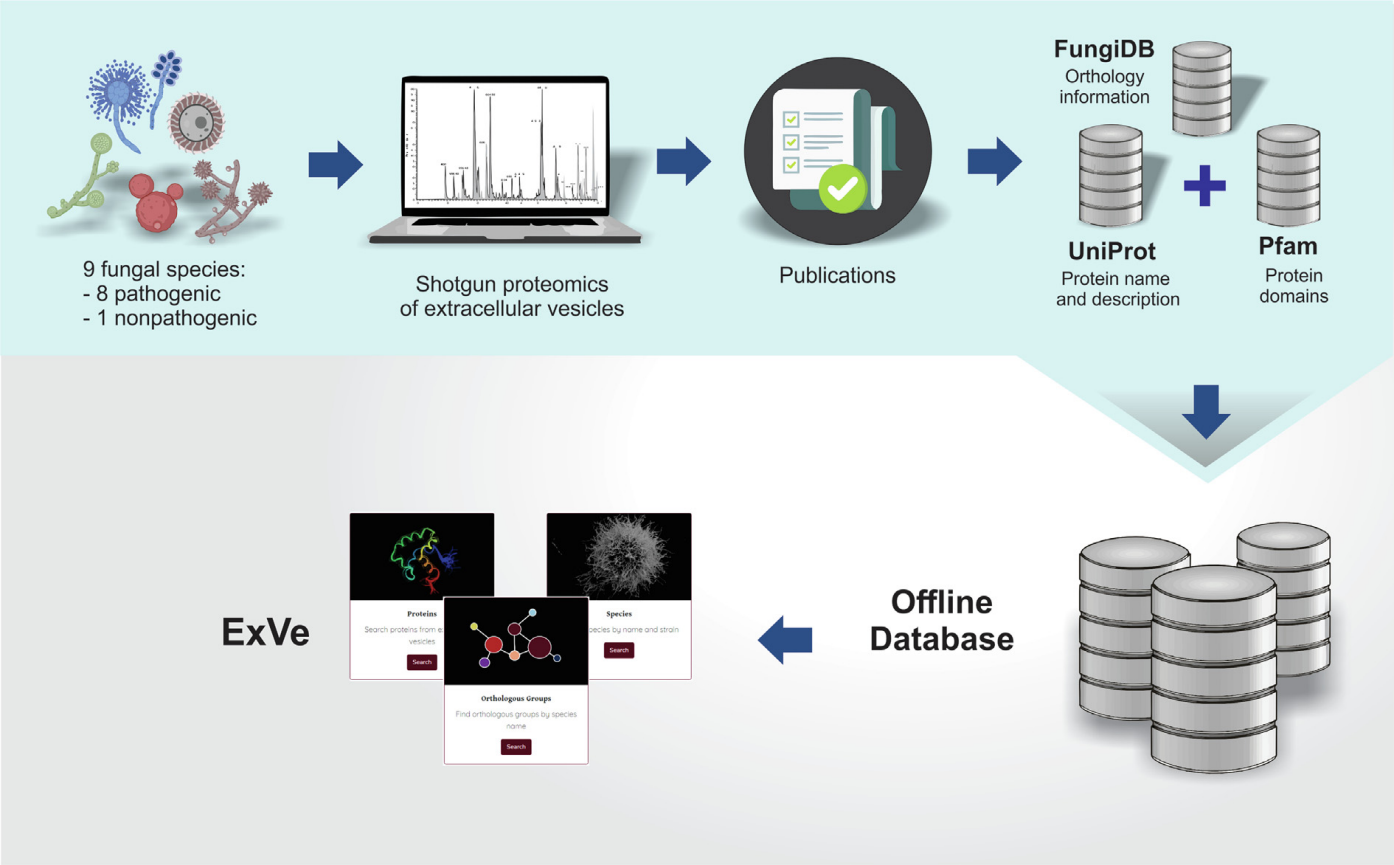
Graphical representation of the ExVe workflow. We retrieved shotgun proteomics data from 9 fungal species (8 pathogenic and 1 nonpathogenic). We performed an association of these data with Uniprot [49], Pfam [48] and FungiDB [46] to provide a web interface with orthology information about proteomic content from fungal EVs.

To build ExVe, first we selected articles with a full description of EV isolation and proteomics methods. From these articles, we used the list of identified proteins by shotgun proteomics experiments available in previously published fungal EV research articles (Fig. 1). We retrieved data from the following eight fungal species with clinical or medical relevance for humans: *Aspergillus fumigatus*, *Candida albicans*, *Cryptococcus deuterogattii, Cryptococcus neoformans*, *Histoplasma capsulatum*, *Paracoccidioides brasiliensis*, *Sporothrix brasiliensis,* and *Sporothrix schenckii*. Additionally, we included *Saccharomyces cerevisiae* as representative of a nonpathogenic fungus (Table 1).

**Table 1:** Related articles for each species of fungi present in ExVe.

Species	Reference
*Aspergillus fumigatus*	[38]
*Candida albicans*	[14], [24], [39], [40]
*Cryptococcus deuterogattii*	[41]
*Cryptococcus neoformans*	[26], [41], [42]
*Histoplasma capsulatum*	[43], [44]
*Paracoccidioides brasiliensis*	[45]
*Sporothrix brasiliensis*	[23]
*Sporothrix schencki*	[23]
*Saccharomyces cerevisiae*	[19]

The aim of ExVe is to offer authors in the field an alternative to access qualitative fungal EV shotgun proteomics identification with focus on orthology. As ExVe relies on third-party analysis, we highlight that each article applied its own statistical threshold, including false positive rates. Proteins with any peptide detected in such experiments are listed as present in EVs from fungal species. We created a computer program written in Perl to retrieve all required data and gather all the information in a single file. First, we retrieved UniProt accession numbers for all proteins based on gene symbols, protein name descriptions and species (Fig. 1) [32]. Thereafter, we used each UniProt accession number to obtain a unique protein name and biological function (protein_description) from UniProt TREMBL and SWISSPROT databases. For protein names, we retrieved the name recommended by the UniProt consortium (“RecName”), and in its absence, the first submitted name (“SubName”) for that protein accession was chosen [32].

### 2.2 Protein orthologous groups

The protein orthologous groups for each protein in our database were retrieved by the OrthoMCL algorithm available from the FungiDB database, release 48 [46] (Fig. 1). We downloaded the web data from all fungal species available at FungiDB [46] to retrieve the orthologous group available to all fungal EV proteins using a dictionary based on UniProt accession number or using the gene symbol associated with a species. Associated with the orthologous group information, we retrieved the most frequent Pfam domain for functional annotation of the group.

### 2.3 ExVe integrated data

In ExVe, users can visualize information about proteins identified in EVs as follows: gene name, protein accession number, protein name, protein description, species, strain, PubMed number, and orthologous group (Table 2). Information about proteins that could not be recovered was named “not available”, and it is available in the downloadable ExVe flat file.

**Table 2:** Data presented on ExVe for proteins present in fungal extracellular vesicles.

Gene Name	UniProt based gene name for proteins [32]
Protein ID	Unique UniProt accession number for proteins [32]
Protein Name	Protein name retrieved from the UniProt consortium [32]
Protein Description	UniProt protein function annotation [32]
Species	Fungi species
Strain	Fungi strain
Type of fungal Culture	Media used for EV isolation (liquid or solid)
Proteomics Method	Equipment used for proteomics analysis
PMID	PubMed number from which the data were obtained [47]
Orthologous Group	Protein orthologous group obtained from the FungiDB [46]
Pfam	The most frequent Pfam ID in each orthologous group (Orthogroup) [48]
Description_pfam	The description of protein domains based on Pfam [48]

### 2.4 Enrichment analysis

**Fig. 2:**
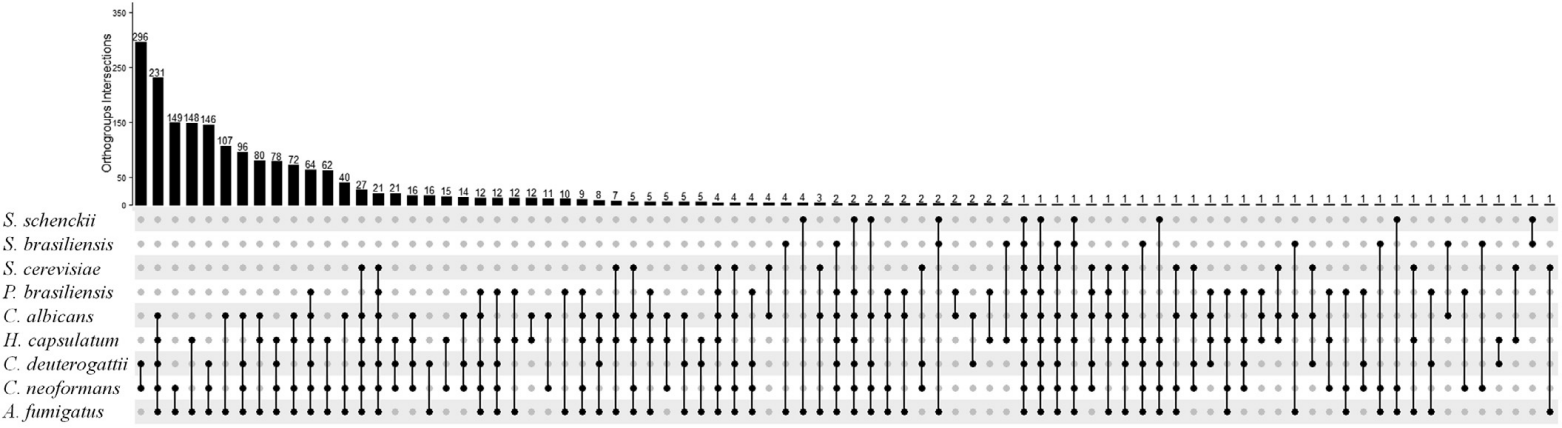
Graphical representation of ortholog group intersections without unique clusters. The upper histogram shows the number of orthologous groups represented in the intersection. The lower dots show the combinations that perform the intersections.

The enrichment analysis of groups of species that shared at least 50 orthologous groups (Fig. 2) was performed using Gene Ontology annotations Fig. 2. Briefly, we retrieved the orthologous groups shared by each group of species and recovered the genes associated with these orthologous groups for a selected representative species. We chose *A. fumigatus* as the reference fungus due to its presence in all groups of species. However, in the group composed exclusively of *Cryptococcus* genus the selected species was *C. neoformans*.

To retrieve Gene Ontology annotations we used FungiDB webtools as in its own guidelines [46]. We retrieved information of biological processes, molecular functions, and cellular components, considering computed and curated evidences with p-value < 0.05. The full list of identifications is available as Supplementary Table 1 (Table S1).

### 2.5 Implementation

ExVe was implemented using the Laravel 5.5 and Docker frameworks in PHP language. A PostgreSQL version 12.3 database was used to store our datasets using Docker containers.

## 3 Results

### 3.1 Proteins identified in fungal EV shotgun proteomics data

In the current version of ExVe, we used protein identifications of shotgun proteomics EVs from *A. fumigatus*, *C. albicans*, *C. deuterogattii, C. neoformans*, *H. capsulatum*, *P. brasiliensis*, *S. brasiliensis, S. schenckii*, and S. *cerevisiae* (Table 3).

**Table 3:** Species, strain, number of distinct proteins, proteomic approach and references used to populate the ExVe database.

Species	Strain	Number of distinct proteins	Proteomics approach	Ref.
*A. fumigatus*	ku80	3,037	EASY-nLC 1000 (Thermo Fisher) Orbitrap Q Exactive Plus (Thermo Fisher)	[38]
*C. albicans*	strain 11* ATCC90028 SC5314 SC5314 YLC280 YLC294 YLC337 YLC344 YLC375 modified SN152	132 50 96 164 242 163 197 213 179 1,110	NanoLC 1D Plus (Eksigent) LTQ XL/ETD linear ion trap (Thermo Fisher) EASY-nLC II (Thermo Fisher) LTQ-Orbitrap Velos (Thermo Fisher) NanoLC 1D Plus (Eksigent) LTQ XL linear ion trap (Thermo Fisher) 1100 nanoflow system (Agilent) LTQ-Orbitrap (Thermo Fisher)	[14], [39], [40], [24]
*C. deuterogattii*	R265	1,292	EASY-nLC 1200 (Thermo Fisher) Orbitrap Q Exactive Plus (Thermo Fisher)	[41]
*C. neoformans*	2ETU-C CAP67 H99 CAP67 K99alpha	26 92 25 111 1847	NanoLC 1D Plus (Eksigent) LTQ XL linear ion trap (Thermo Fisher) NanoLC 1D Plus (Eksigent) LTQ XL linear ion trap (Thermo Fisher) EASY-nLC 1200 (Thermo Fisher) Orbitrap Q Exactive Plus (Thermo Fisher)	[26], [42], [41]
*H. capsulatum*	ATCC26032	283	NanoLC 1D Plus (Eksigent) LTQ XL linear ion trap (Thermo Fisher)	[43], [44]
*H. capsulatum (cont.)*	ATCCG217B	1,127	Ekspert nanoLC 400 (Eksigent) 5600 TripleTOF (AB Sciex)	[44]
*P. brasiliensis*	Pb18	205	NanoLC 1D Plus (Eksigent) LTQ XL linear ion trap (Thermo Fisher)	[45]
*S. brasiliensis*	5110	63	NanoLC-1DPlus (Proxeon) LTQ-Orbitrap (Thermo Fisher)	[23]
*S. schenckii*	M−64	40	NanoLC-1DPlus (Proxeon) LTQ-Orbitrap (Thermo Fisher)	[23]
*S. cerevisiae*	RSY113 RSY255 RSY782 RSY954 SEY6210 SF264-1D Snf7 VPS23	88 102 96 103 99 99 91 110	nanoLC 1D Plus (Eksigent) LTQ XL/ETD linear ion trap (Thermo Fisher)	[19]
*male patient at Institute of Hematology Arthur Siqueira Cavalcanti				

The EV isolation methods of eleven out of the thirteen articles selected to populate ExVe were based on the original study on fungal EVs using liquid media [8], [50]. The exceptions were the *C. albicans* study by Zarnowski and colleagues (2018) using biofilms [24] and the study by Rizzo et al. on *C. neoformans* and *C. deuterogattii* performing EV isolation from solid media [29].

ExVe integrates data for 11,433 hits detected in fungal EVs. Any identified protein in each study with no UniProt accession number was purged from ExVe. We identified 5,367 proteins with a SwissProt [32] accession number, of which 1,879 have a biological function annotated. For the remaining proteins, 6,043 were associated with a Trembl accession number, of which 614 have computationally inferred biological functions [32].

We noticed that different sets of equipment were used to generate the proteomics shotgun data. The NanoLC 1D Plus (Eksigent) associated with LTQ XL linear ion trap (Thermo Fisher) was the most frequent combination to generate shotgun proteomics data, which was applied to *S. cerevisiae, P. brasiliensis, H. capsulatum, C. neoformans* and *C. albicans*. The spectrometer Orbitrap Q Exactive Plus (Thermo Fisher) associated to the chromatograph EASY-nLC (1000/1200) (Thermo Fisher) was used to generate shotgun proteomics data of *A. fumigatus*, *C. albicans* and *C. neoformans*. Interestingly, the spectrometer 5600 TripleTOF (AB Sciex) in conjunction with chromatograph Ekspert nanoLC 400 (Eksigent) enabled the identification of more than 1,100 proteins for each *H. capsulatum* study, the highest number of detected proteins.

### 3.2 Protein orthologous groups

ExVe has 11,189 proteins clustered in 3,834 different ortholog groups (Table 4), whereas 292 were defined as orphan proteins, which are unavailable for website visualization.

**Table 4:** Number of orthologous groups for each species at ExVe.

Species	Number of orthologous groups
*A. fumigatus*	2,717
*C. albicans*	1,019
*C. deuterogattii*	1,194
*C. neoformans*	1,736
*H. capsulatum*	1,084
*P. brasiliensis*	186
*S. brasiliensis*	38
*S. schenckii*	30
*S. cerevisiae*	95

Our findings indicate that *A. fumigatus* and *C. neoformans* presented the highest number of unique orthologous groups: 1,253 and 590, respectively. The orthologous group OG6_100083 was unique to all nine species in ExVe. This orthologous group comprises a set of chaperone-encoding genes with the Hsp70 protein (PF00012) as the most frequent Pfam protein domain. The comparison of all nine fungi revealed that *C. neoformans* and *C. deuterogattii* shared 296 orthologous groups, which was the highest number among all possible pairs of species (Fig. 2). However, *C. albicans*, *H. capsualtum*, *C. deuterogattii*, *C. neoformans* and *A. fumigatus* was the group of different genera that shared the highest number of orthologous groups (231 groups).

We used FungiDB tools to retrieve Gene Ontology (GO) annotations to investigate the enrichment of GO terms for species that shared more than 50 orthologous groups (Table S1). With the highest number of orthologous groups shared, the *Cryptococcus* genus had an “extracellular region” term (GO:0005576) enriched for the “cellular compound” category (Table S1). Additionally, “hydrolase activity” terms (GO:0016810, GO:0004553, GO:0016798) were enriched in the “molecular function” category (Table S1). The “biological process” category in the *Cryptococcus* genus contained the “mRNA processing” and “mRNA splicing” associated terms (GO:0000375, GO:0000398, GO:0000377, GO:0008380, GO:0006397, GO:0016071) were the most enriched GO terms.

The second group of species with the highest number of orthologous groups shared (*C. albicans*, *H. capsualtum*, *C. deuterogattii*, *C. neoformans* and *A. fumigatus*) revealed the “cytoplasm” term (GO:0005737) as the most enriched GO term for the “cellular component” category. In this group, the “organonitrogen compound biosynthetic term” (GO:1901566) was the most enriched “biological process” term. At last, our analysis revealed the enrichment of “structural” terms (GO:0005198 and GO:0003735) for the “molecular function” category for this group of species (Table S1).

Next, we investigated the occurrence of orthologous groups related to fungal pathogenicity. According to our analysis, no orthologous group was exclusively present in the eight pathogenic fungi (Table 5). However, if a single pathogenic species is excluded at a time from the analysis, some orthologous groups are pinpointed. If a given *Sporothrix* genus was excluded at a time from the comparison to all other fungi, another exclusive pathogenic orthologous group was identified. *S. brasiliensis* exclusion permitted the identification of OG6_100304 and OG6_100832 exclusive pathogenic orthogoups, which represent proteins associated with nucleoside-diphosphate kinase (PF00334) and ribosomal S17 (PF00833) Pfam domains, respectively. If *S. schenckii* was not considered in the comparison, the orthologous groups OG6_100082 and OG6_100425 are identified, which are associated with core histone H2A/H2B/H3/H4 (PF00125) and RNA recognition motif (PF00076) Pfam protein domains, respectively. The investigation of orthologous groups exclusively detected in the nonpathogenic *S. cerevisiae* fungus unveiled the following orthologous groups with protein domains according to Pfam: OG6_100674, OG6_102300, OG6_142972, OG6_500194, and OG6_222591. These orthologous groups are related to dihydroorotate dehydrogenase (PF01180), the phosphoadenosine phosphosulfate reductase family (PF01507), glucanosyltransferase (PF03198), and the glycolipid 2-alpha-mannosyltransferase (PF01793) Pfam protein domains. The orthologous group OG6_222591 was identified as exclusive to *S. cerevisiae*, but no Pfam protein domain is available yet.

**Table 5:** Most frequent in ExVe and unique *S. cerevisiae* ortholog groups.

Ortholog group	Protein Domain*	*C. neoformans*	*C. deuterogattii*	*A. fumigatus*	*C. albicans*	*H. capsulatum*	*S. schenckii*	*S. brasiliensis*	*P. brasiliensis*	*S. cerevisiae*
OG6_100083	Hsp70 protein	●	●	●	●	●	●	●	●	●
OG6_100304	Nucleoside diphosphate kinase	●	●	●	●	●	●		●	
OG6_100832	Ribosomal S17	●	●	●	●	●	●		●	
OG6_100082	Core histone H2A/H2B/H3/H4	●	●	●	●	●		●	●	
OG6_100425	RNA recognition motif. (a.k.a. RRM, RBD, or RNP domain) **	●	●	●	●	●		●	●	
OG6_100674	Dihydroorotate dehydrogenase									●
OG6_102300	Phosphoadenosine phosphosulfate reductase family									●
OG6_142972	Glucanosyltransferase									●
OG6_500194	Glycolipid 2-alpha-mannosyltransferase									●
OG6_222591	No domain identified									●

*Protein domain is the most represented Pfam domain in the orthologous group from FungiDB.

**a.k.a = also known as.

### 3.3 Online application, visualization module and functionalities

**Fig. 3:**
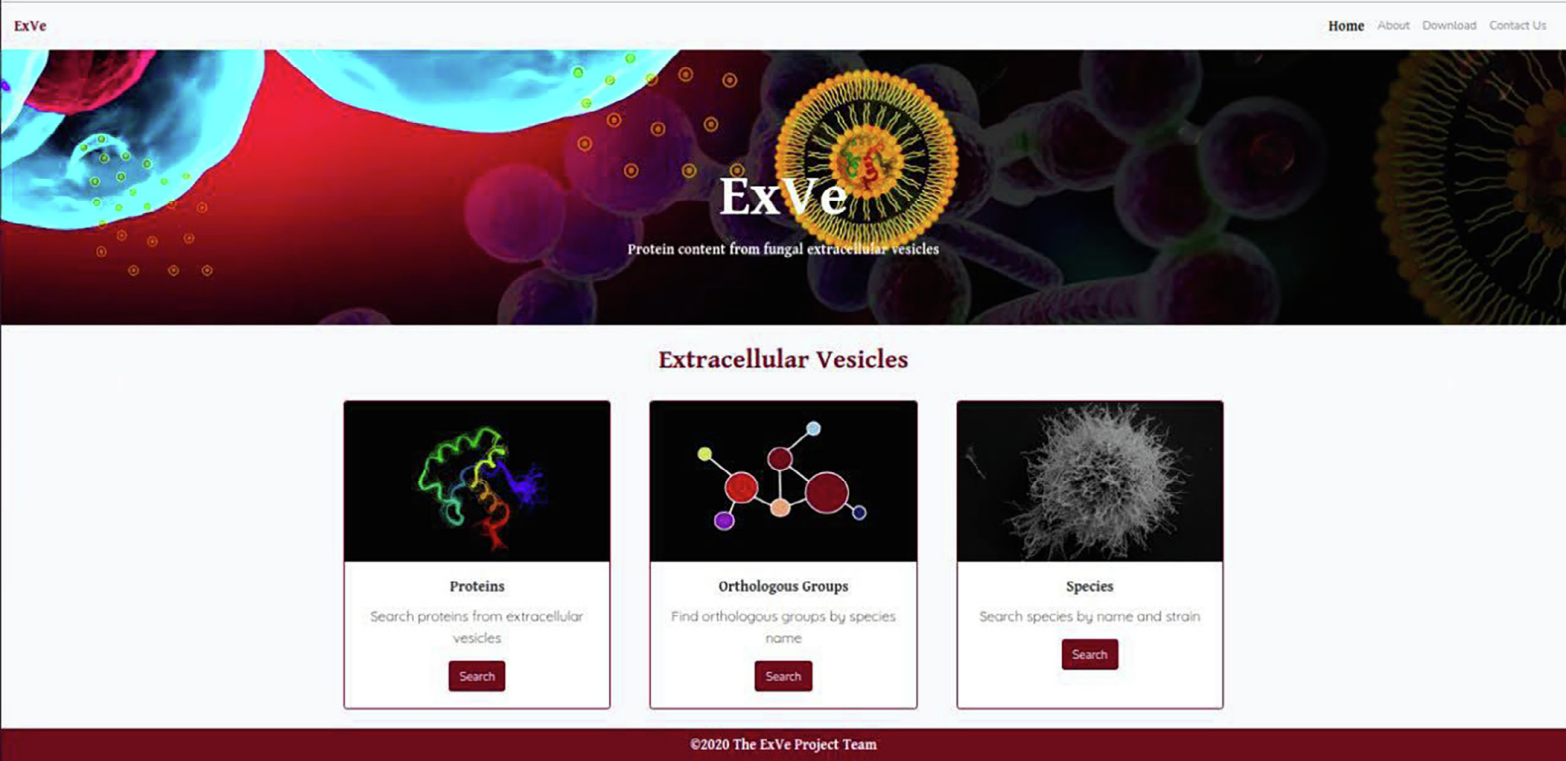
The graphical interface of the ExVe database. A total of 6 menus for different functionalities are implemented in the database.

The online application is available under the URL http://exve.icc.fiocruz.br. The ExVe contains 6 menus, named “About”, “Contact Us”, “Download”, “Species”, “Orthologous Groups”, and “Proteins” (Fig. 3). The “About” menu contains a brief description of the ExVe database, in addition to listing some features of the web system. The “Contact Us” menu displays the mail contact for questions, error reports, feature requests and dataset proposals. The “Download” menu allows the user to download the ExVe database.

**Fig. 4:**
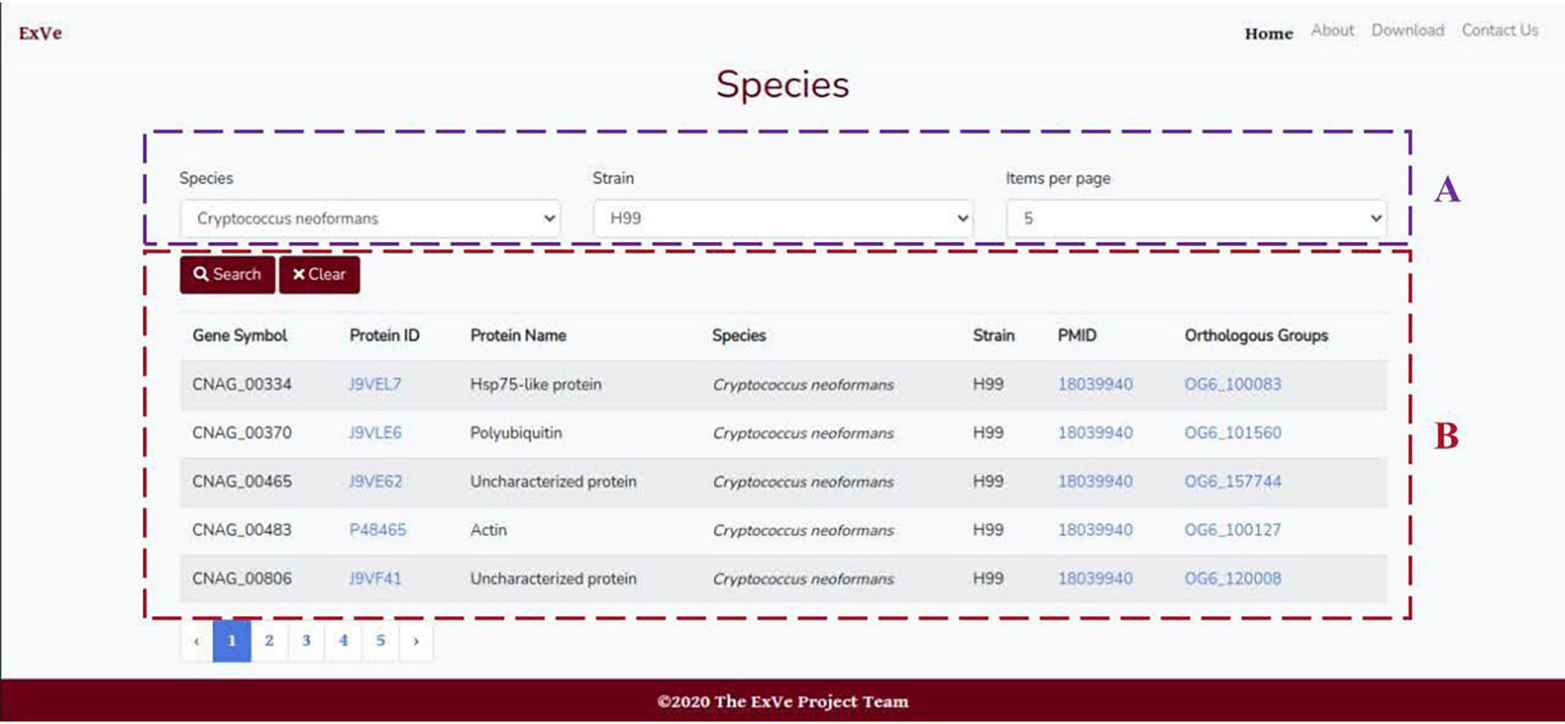
(A) Input parameters for Species menu. Species, strain (optional) and items per page. (B) Output for *C. neoformans* (strain H99) search.

In the “Species” menu (Fig. 4), the user can select the organism species and strain from the complete list (currently including 9 species and 28 strains, as listed in Table 3)**.** For a user-specified species and/or strain, all available gene symbols, UniProt protein IDs and names, strains, orthologous groups and PubMed links [47] are displayed in a table.

**Fig. 5:**
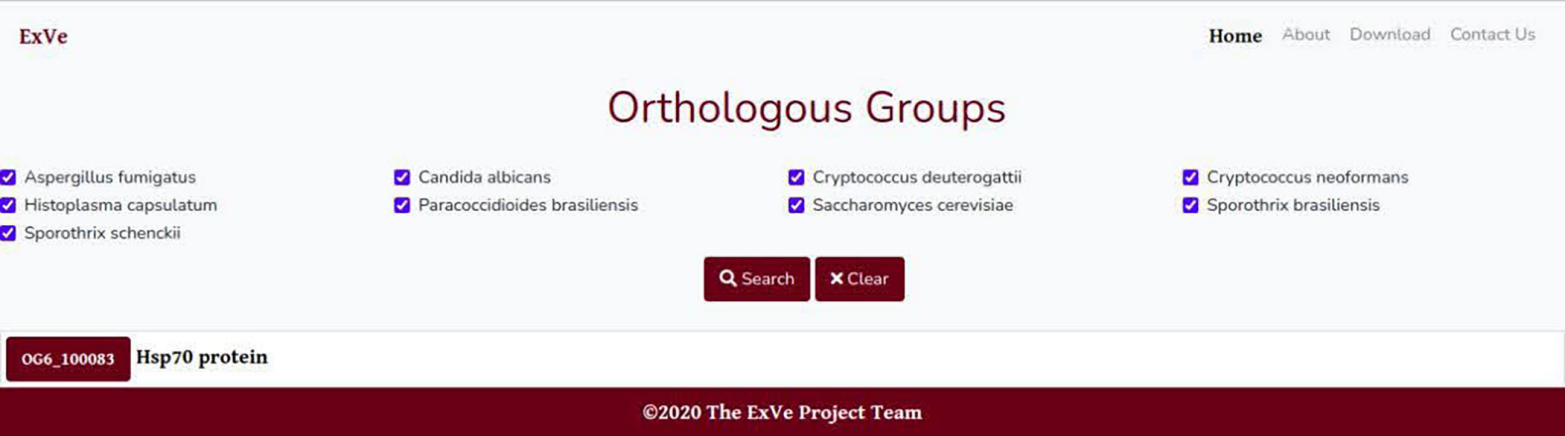
ExVe graphical output for all species search. The ‘‘Orthogroups” menu contains various widgets, which are designed to receive user input.

The “Orthogroups” menu provides features to visualize all available orthologous groups and PFAM domains they are associated with (see Fig. 5). Users can submit one or more species and receive all groups to which both have orthologous proteins. When a protein is not assigned to any group of orthologs, it is grouped in a cluster termed “not available”.

**Fig. 6:**
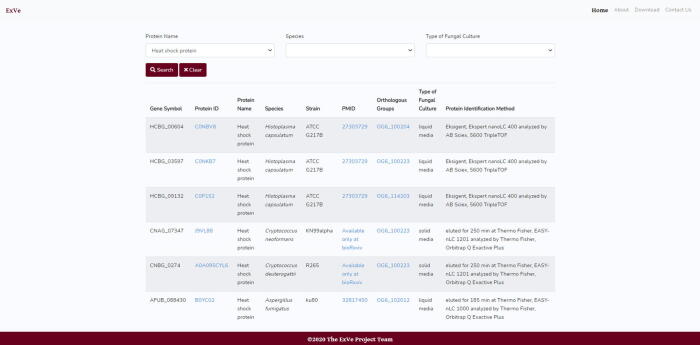
Input parameters for Proteins menu. Output for Heat shock protein search.

The “Proteins” menu provides two principal functionalities (Fig. 6). Users can search for a given protein, and ExVe returns all orthologous groups with which the selected protein is associated, the gene symbol when available, the species and strain from which each protein was isolated, protein name, PMID, and the UniProt ID [32]. The UniProt ID field contains a link where users can access additional protein information such as the name and description of the protein. Additionally, on this page, the user can be redirected to the research article that described the protein by clicking on the PMID field [47]. Another feature on this page is the redirection to the UniProtKB consortium page [32] when clicking on the UniProt ID.

**Fig. 7:**
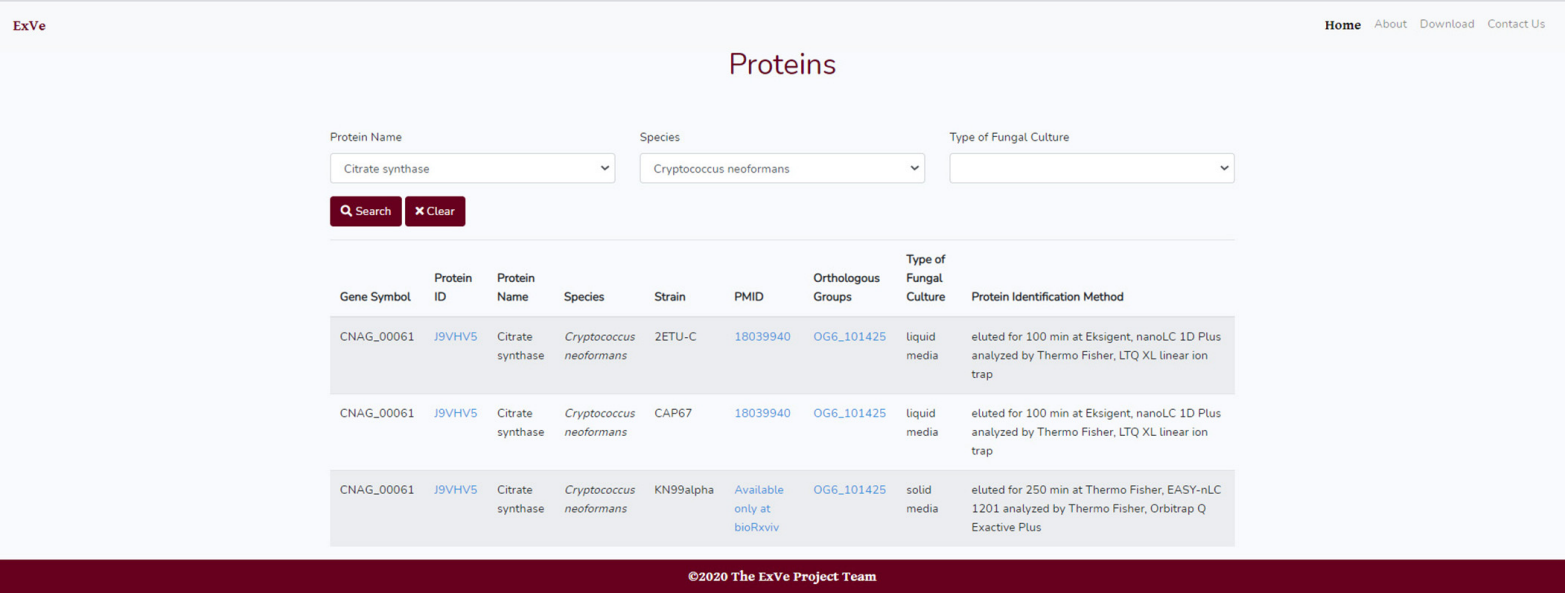
Input parameters for Proteins menu. Output for citrate synthase and *Cryptococcus neoformans* search.

Another main feature of the menu is the possibility of filtering by protein, species name and type of fungal culture (liquid or solid media), and ExVe returns the same information listed above (Fig. 7).

## 4 Discussion

Fungal EVs have been recently proposed as vaccine candidates [51], [52], but the molecules inducing protection of different hosts remain unknown [41], [51]. Recently, we identified an EV peptide inducing protection in an invertebrate host [53], but the immunological roles of vesicle-associated proteins are still unclear. This scenario might be linked to one major gap in the field of fungal EVs: the lack of well-characterized biomarkers, as well as molecules with potential to be vaccine candidates. In this context, a systematic analysis of components of fungal EVs produced by different species will likely benefit the whole field. Moreover, an orthology analysis of EV components produced by pathogenic species may contribute to guide future research on the functions of vesicular molecules.

Reference EV databases such as Vesiclepedia [34] and Exocarta [33] have limited data regarding fungal species, while other eukaryotic and prokaryotic organisms have abundant information. We are launching ExVe as a free web repository that integrates EV proteins previously identified by shotgun proteomics into nine fungal species using orthology data, with possibility of expansion depending on the availability of data in the future literature. The use of orthology information can improve comparative studies and assist in protein function prediction [54]. Although there are orthology databases such as FungiDB [46], OrthoMCL [37], and AYbRAH [31], none of them provide a subset of fungal EV proteomics data. Additionally, the association of ExVe with FungiDB allows users to assess a large amount of biological data and bioinformatic resources to improve data analysis [46].

Distinct protocols for EV protein analysis will influence the number of identified proteins [27], [30]. The variability in the EV isolation methods, mostly based on differential ultracentrifugation of samples obtained from liquid media being, has the same potential to affect protein detection [8], [27], [50]. Alternative protocols of isolation of fungal EVs, including chromatographic separation and the use of solid media, have been recently described [9], [27], [29], [30]. All articles used in to populate the ExVe databases analyzed fungal EVs by differential ultracentrifugation from liquid media [8], [24], [50], excepting for the recent study of Rizzo and colleagues [41].

The peptide identification criteria may also affect the quantity and quality of proteins identified in fungal EVs [28], [55]. Nevertheless, a restricted number of studies in ExVe overpassed the number of 1,000 proteins identified. Moreover, with the exceptions of A. fumigatus [38] and C. deuterogattii [41] that have an unique study for each specie, there were different studies with different proteomics approaches to detect proteins from EVs (Table 3). Therefore, our data suggests that the different proteomics approaches had a limited impact on the identification of orthologous groups in fungal species.

According to our analysis, OG6_100083 is the only ortholog group in all species available at ExVe. This ortholog group gathers sequences of heat shock proteins, with a focus on the HSP70 family Pfam protein domain [46]. Several studies imply the high sequence conservation of this protein family among different groups and species [56], [57]. The intracellular role of Hsp proteins as chaperones and in the modulation of stress response was previously described [58], [59]. HSP70 proteins were identified at the extracellular environment playing multiple roles [59], [60]. Notably, Hsp70 was characterized as a component of fungal EVs [59]. The functions of extracellular Hsp70 can be numerous, but they could be associated with cell signaling mainly by the modulation of macrophage activation, and attenuation of the immune response [58], [61].

In fungi, heat shock proteins have been described in the stress response and survival of different fungi under adverse environmental conditions such as temperature changes, starvation and antifungal stress [56], [57], [62]. Heat shock proteins are also involved in morphological changes, including the transition from mycelium to yeast, and have an important role in the interaction with host immune cells [56], [63]. Indeed, heat shock proteins are apparently essential for the formation of fungal EVs. In *H. capsulatum*, binding of antibodies raised against heat shock proteins to the fungal surface resulted in EVs with altered protein loading [44]. In this fungus and in *C. neoformans*, these proteins were recognized by antibodies produced by infected patients [14], [26]. In summary, our results and the recent literature strongly suggest heat shock proteins as main components of fungal EVs.

Other 4 orthologous groups were identified in 7 of 8 fungal pathogenic species, but not in *S. cerevisiae* (Table 5). The nucleoside diphosphate kinase (Pfam ID PF00334) is an Pfam protein domain exclusively identified in most pathogenic fungi. This protein domain has already been related to EVs derived from breast cancer, and associated [64]. In fungi, this protein domain is potentially associated with fungal resistance to oxidative and thermal stress [65]. For instance, the gene encoding the nucleoside diphosphate kinase was shown to be essential in *A. flavus*, with a role in spore production and sclerotia formation [66], [67]. Interestingly, disruption of this gene in *S. cerevisiae* resulted in unaltered growth ratio and spore production levels [68]. Because this orthologous group was identified only in EVs produced by pathogenic species, we speculate that it plays a role in pathogenicity.

Histones have a central function in eukaryotes, controlling chromatin accessibility and chromosome segregation during mitosis. In *C. albicans*, histone H2A was described to regulate aneuploidy, argued as a strategy to acquire tolerance to antifungal therapy [69]. H3 and a variant histone protein were described to play a role in biofilm and planktonic forms of *C. albicans*, depicting the relevance of such proteins in the fungal cell cycle [70]. Regarding the role of histones in fungal pathogenicity, the histone acetyltransferase Gcn5 was shown to work in chromatin remodeling in response to stress induced by the human host environment during *C. neoformans* invasion [71]. Although the biological function of histones is known to be related to chromatin accessibility, additional functions could be found in such proteins. Using a mouse model, an H2B-like protein was detected in the *H. capsulatum* cell surface, which was used to investigate its role in the immune response [72]. Macrophage histones were detected at the surface of EVs, which was associated with proinflammatory responses [73]. However, the biological function of fungal histones in EVs is not yet known, we suppose these proteins could be associated with virulence strategies since they were not detected in *S. cerevisiae*. The fact that a histone-like protein was detected on the cell surface in one of the pathogenic fungi studied here sheds light on the reason why such proteins were identified in EVs.

EVs contain distinct types of molecules in their lumen, including nucleic acids and proteins. Therefore, it is expected to find proteins holding an RNA binding motif that would carry RNAs, an unstable molecule. mRNA trafficking occurs extensively in the cytosol of several fungi [74], [75]. In EVs, short RNAs were detected in *C. neoformans*, *P. brasiliensis, C. albicans*, and *S. cerevisiae* [76]. Surprisingly, according to our analysis, the RNA recognition motif (Pfam ID PF00076) was identified only in pathogenic species, not in *S. cerevisiae*. This observation should be investigated in depth in further studies to confirm that such proteins may act carrying some specific pathogenic RNAs.

Some of the orthologous groups were exclusively identified in *S. cerevisiae,* and not in any fungal pathogenic species. This observation points to the existence of still unknown differential mechanisms of protein loading into pathogenic and nonpathogenic fungal EVs. The dihydroorotate dehydrogenase Pfam protein domain (Pfam ID PF01180), exclusively found in the *S. cerevisiae* EVs, is associated with key proteins related to fungal pyrimidine biosynthesis, with investigations regarding its structure and mechanism of action in *S. cerevisiae* [77]. The phosphoadenosine phosphosulfate reductase family (Pfam ID PF01507), also exclusive to *S. cerevisiae* EVs, is another Pfam protein domain associated with essential fungal metabolism. These proteins are vital to sulfur uptake by the action of 3′-phosphoadenosine-5′-phosphosulfate (PAPS) reductases. This class of fungal enzymes was proposed to be the target of new molecules aiming at fungal therapy, with a focus on *Aspergillus* species [78], [79].

Fungal EVs have been recently linked to the formation of the cell wall [38], [80], a key component of the fungal cell. These reports agree with our findings showing an association of fungal EVs and the cell wall. Glycolipid 2-alpha-mannosyltransferase (Pfam ID PF01793) is responsible for the mannosylation of the lipid-linked oligosaccharide, which is required for the formation of *O*-linked saccharides during cell wall synthesis [81]. Fungal proteins with the Pfam protein domain glucanosyltransferase (Pfam ID PF03198) have the β-1,3-glucanosyltransferase (Gas1) operate in cell wall synthesis, silencing of rDNA expression, and stress response [82], [83]. Since Gas1 was detected in EVs produced by yeast cells and not in *A. fumigatus,* the existence morphology-related functions for this EV protein are expected.

## 5 Conclusions

The current purpose of ExVe was to integrate available EV proteins identified by shotgun proteomics data for medically relevant fungal species. However, a database that includes data for different molecules could contribute more to the elucidation of additional questions [35]. Therefore, we plan to implement data recovery from other molecules such as lipids and RNAs, aiming for ExVe improvement. Additionally, we aim to enable sequence search, gene ontology and network analysis as future perspectives. Our proposal is that ExVe will be updated annually with newly available data. ExVe is open to continuously integrating proteins identified by shotgun proteomics data from the scientific community.

## Appendix A Supplementary data

Supplementary data 1 Gene Ontology information (biological process, molecular function and cellular compound) about the fungal groups that shared more than 50 orthologous groups. Each sheet represents the information of each group. The species name is represented in this table by the first capital letter of the genus followed by the first three letters of the species in lowercase.
